# Effect of Alkali-Acid-Heat Chemical Surface Treatment on Electron Beam Melted Porous Titanium and Its Apatite Forming Ability

**DOI:** 10.3390/ma8041612

**Published:** 2015-04-08

**Authors:** Suzan Bsat, Saber Amin Yavari, Maximilian Munsch, Edward R. Valstar, Amir A. Zadpoor

**Affiliations:** 1 Department of Mechanical and Aerospace Engineering, Carleton University, 1125 Colonel by Drive, Ottawa, ON K1S 5B6, Canada; 2 Faculty of Mechanical, Maritime, and Materials Engineering, Delft University of Technology, Mekelweg 2, 2628 CD Delft, The Netherlands; E-Mails: S.AminYavari@tudelft.nl (S.A.Y.); E.R.Valstar@lumc.nl (E.R.V.); a.a.zadpoor@tudelft.nl (A.A.Z.); 3 Implantcast GmbH, Lueneburger Schanze 26, D-21614 Buxtehude, Germany; E-Mail: m.munsch@implantcast.de; 4 Department of Orthopaedics, Leiden University Medical Center, Albinusdreef 2, 2333 ZA Leiden, The Netherlands

**Keywords:** porous titanium, chemical surface treatment, apatite formation, additive manufacturing

## Abstract

Advanced additive manufacturing techniques such as electron beam melting (EBM), can produce highly porous structures that resemble the mechanical properties and structure of native bone. However, for orthopaedic applications, such as joint prostheses or bone substitution, the surface must also be bio-functionalized to promote bone growth. In the current work, EBM porous Ti6Al4V alloy was exposed to an alkali acid heat (AlAcH) treatment to bio-functionalize the surface of the porous structure. Various molar concentrations (3, 5, 10M) and immersion times (6, 24 h) of the alkali treatment were used to determine optimal parameters. The apatite forming ability of the samples was evaluated using simulated body fluid (SBF) immersion testing. The micro-topography and surface chemistry of AlAcH treated samples were evaluated before and after SBF testing using scanning electron microscopy and energy dispersive X-ray spectroscopy. The AlAcH treatment successfully modified the topographical and chemical characteristics of EBM porous titanium surface creating nano-topographical features ranging from 200–300 nm in size with a titania layer ideal for apatite formation. After 1 and 3 week immersion in SBF, there was no Ca or P present on the surface of as manufactured porous titanium while both elements were present on all AlAcH treated samples except those exposed to 3M, 6 h alkali treatment. An increase in molar concentration and/or immersion time of alkali treatment resulted in an increase in the number of nano-topographical features per unit area as well as the amount of titania on the surface.

## 1. Introduction

Titanium has long been used for biomedical applications because of its combined superiority in biocompatibility, mechanical properties and corrosion resistance [1,2,3]. However, orthopaedic implants fabricated of solid titanium and its alloys have been shown to lack in performance due to the stress shielding effect, subsequently causing implant loosening [4,5]. Although titanium has a lower Young’s modulus than alternative biomaterials (*i.e.*, stainless steel or cobalt-chrome), stress shielding continues to exist causing bone resorption due to the difference in stiffness between the titanium implant (110 GPa for alloy Ti6Al4V) and adjacent bone (20–30 GPa for cortical bone) eventually initiating implant loosening [6]. Porous titanium was thus developed with an aim to improve implant performance by reducing the difference in stiffness between the interacting implant and adjacent bone through the addition of pores, in some cases achieving implant stiffness between 3.5–25 GPa [6,7,8]. Ideally the porous titanium is to mimic native bone structure and stiffness to eradicate bone resorption. Furthermore, porous titanium has also improved implant performance by increasing surface area and porosity allowing higher levels of bone ingrowth [9,10] and the incorporation of functional molecules [11]. Over the past decade, porous titanium has thus emerged as a breakthrough material showing potential by minimizing stress shielding effects, improving bone ingrowth and creating larger surface areas for drug delivery media, particularly in orthopaedics for joint prosthesis [12,13,14,15] or bone substitution [16,17,18,19]. 

New ways to manufacture porous titanium are constantly being developed with an aim to achieve similar porous structure and mechanical properties as native bone. As a result, the porous structure will allow for bone ingrowth and incorporation of functional molecules while the mechanical properties are important in avoiding stress shielding effects. Various manufacturing methods such as gel casting [6], loose powder sintering [7], powder metallurgy space holder and titanium fibre sintering [8] have been successfully developed. Yang *et al.* [6] demonstrated that with gel casting methodologies porosities between ~38%–58% resulted in specific Young’s moduli between 7–25 GPa, fitting within the range of native cortical bone stiffness. In a comparison study between sintering and space holder methodologies, loose powder sintering formed an interconnected structure with ~42% porosity with specific Young’s moduli of 20–25 GPa however the space holder technique dominated as the size of pores and porosity were controllable achieving better mechanical properties [7]. A porosity between 50%–70% with specific Young’s moduli between 3.5–4.2 GPa was obtained for porous titanium fabricated by titanium fibre sintering, a potential candidate for cancellous bone substitution [8].

Several manufacturing methods have clearly demonstrated their abilities in achieving porosities and mechanical properties close to that of native bone, however, despite their achievement they are limited to a range of pore sizes and porosities and to their control over the final structure [6,7,8]. Advanced additive manufacturing techniques offers the precision and control over pore size and distribution, surface area and micro-architecture that cannot be matched by other manufacturing methods [20,21,22,23,24,25]. Advanced additive manufacturing techniques, such as electron beam melting (EBM), can therefore produce highly porous metallic structures with precisely controlled micro-architectures. With such a controlled method, structures can be fabricated to consist of varying porous micro-architectures allowing manipulation over the distribution of mechanical properties throughout the implant subsequently controlling the load bearing distribution throughout the structure. Furthermore, with advanced additive manufacturing the highest levels of porosity can be achieved further increasing space for more bone ingrowth [9,10] or surface area for drug delivery media [11].

Although high porosity, ideal mechanical properties and structure can be obtained through advanced additive manufacturing techniques, porous titanium structures must also be bio-functionalized to aid bone growth and integration. Several surface treatments such as plasma spray [26], gelatin [27], anodization [28] and chemical [28,29,30] treatments have been applied to porous titanium to improve its bio-functionality. Chemical surface treatments in particular have been successful in transforming titanium and titanium alloy surfaces from biologically inert to bio-functionalizing surfaces and are desirable due to their ease of application and low cost [28,29,30,31]. More specifically, alkali-acid-heat (AlAcH) treatment is a promising candidate among chemical treatments as it has been shown to effectively bio-functionalize the surface of porous titanium by creating nano-topographical features and modifying the surface chemistry of the structure [28,31] while maintaining adequate mechanical properties [32]. Since the surface properties of porous titanium are highly dependent on manufacturing technique, the effects of AlAcH treatment differs for each case, however, Takemoto *et al.* [31], successfully demonstrated promising morphology, apatite formation and bone regeneration for porous titanium fabricated by plasma spray. Amin Yavari *et al.* [28] showed similar results for porous titanium fabricated by selective laser melting (SLM). 

The current work evaluates the use of AlAcH treatment to bio-functionalize the surface of porous titanium alloy Ti-6Al-4V fabricated by EBM by examining its apatite forming ability. Various molar concentrations (3, 5, 10M) and immersion times (6, 24 h) of the alkali treatment were used for the AlAcH treatment to determine optimal parameters. Following AlAcH treatment, the apatite forming ability of the samples were evaluated using simulated body fluid (SBF) immersion testing. The micro-topography and surface chemistry of AlAcH treated porous titanium samples were examined before and after immersion in SBF using scanning electron microscopy (SEM) and energy dispersive X-ray spectroscopy (EDS). 

## 2. Results and Discussion

In the current work, porous titanium fabricated by EBM was AlAcH treated to produce nano-topographical features and a crystalline titania layer to stimulate the formation of Ca and P, with a final objective of improving apatite forming ability. As shown in previous studies, the formation of nano-topographical features [33,34,35] and the formation of crystalline titania [28,31] helps stimulate the formation of Ca and P, apatite and bone.

### 2.1. AlAcH Treatment

**Figure 1:**
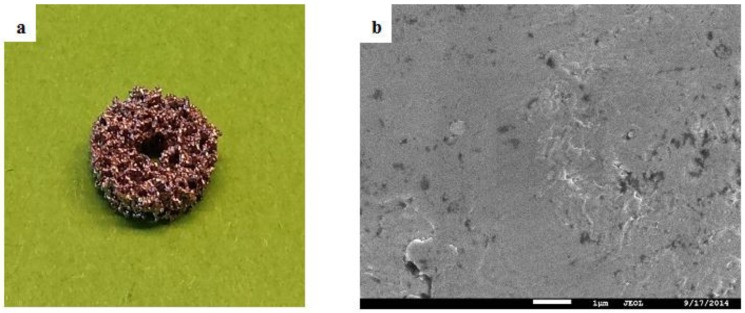
(**a**) Macrograph of test sample and (**b**) SEM micrograph of AsM surface.

**Figure 2:**
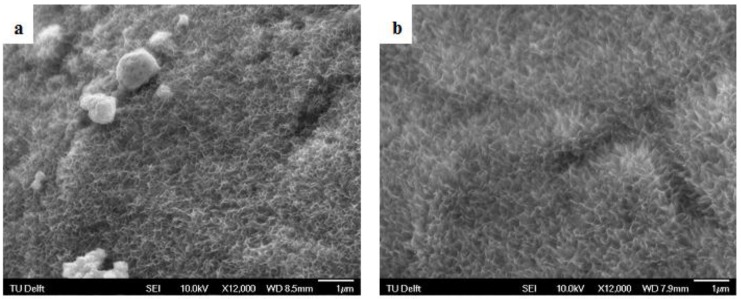

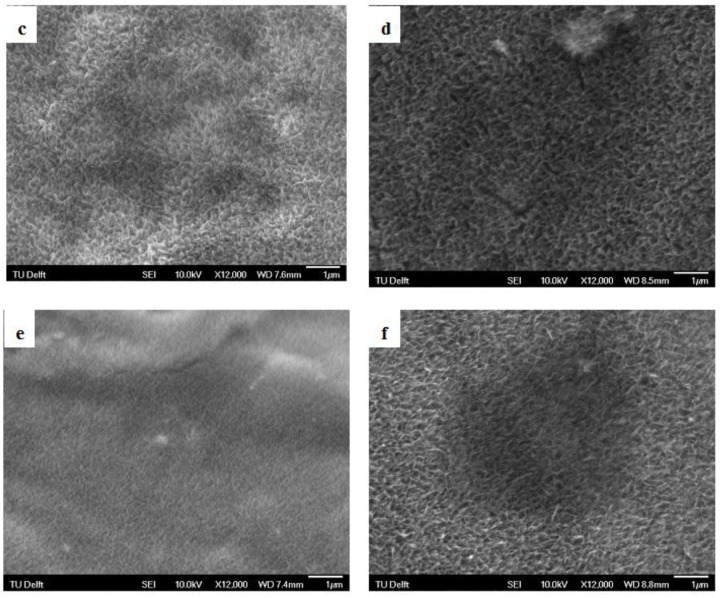
SEM micrographs of samples after AlAcH treatment for various alkali parameters (**a**) 3M, 6 h (**b**) 3M, 24 h (**c**) 5M, 6 h (**d**) 5M, 24 h (**e**) 10M, 6 h and (**f**) 10M, 24 h.

SEM analysis of the AlAcH treated samples revealed modified surfaces with irregular nano-topographical features ranging between 200 and 300 nm in size as compared to the smooth and featureless AsM surfaces (Figure 1 *vs.* Figure 2). The same topographical transformation was observed for all alkali molar concentrations and immersion times, however, those samples treated with stronger molar concentrations had increased nano-topographical features per unit area. Previous studies have shown that the presence of nano-topographical features helps promote the initial formation of Ca and P, and later apatite or bone [33,34,35]. The natural human extracellular matrix is a complex network made up of a collection of nanoscale structures and features and hence is stimulated and interacts with cells on a nanoscale level [36,37,38]. For bone, the use of artificial nanostructures allows intimate interactions with the first level of bone structural hierarchy allowing repopulation and re-synthesis of a new matrix for bone. Based on this understanding of the significance of nano-features, the current results suggest that an AlAcH treatment with higher molar concentration is ideal as stronger molar concentrations of NaOH resulted in increased nano-topographical features per unit area. This may theoretically provide more opportunity for interaction between the implant surface and cells. 

During the chemical treatment, immersion in NaOH alkali solution introduces a sodium titanate layer, the sodium is then removed by HCl acid leaving behind a layer of amorphous titania [31]. The heat treatment then transforms the titania from amorphous to crystalline (anatase and rutile), encouraging the formation of apatite because of its favorable atomic arrangement. Following AlAcH treatment, EDS elemental analysis confirmed a modified surface chemistry composed predominantly of oxygen and titanium as compared to the AsM samples composed primarily of titanium alone (Table 1 *vs.* Table 2). This was expected as the formation of crystalline titania is the culminating result of the AlAcH treatment [31]. Similar surface chemistry was observed for all AlAcH treated samples exposed to various alkali molar concentrations and immersion times, however, in general those exposed to higher concentrations and/or longer immersion times had increased oxygen content suggesting a more prominent titania layer (Table 2). This can be explained by the samples’ exposure to increased concentrations and/or immersion times of NaOH allowing more sodium titanate to form which eventually forms the final crystalline titania layer [31]. The increased amount of oxygen observed for the 3M, 6 h sample is assumed to be an outlier based on the trend of increased oxygen content with increased concentration and/or immersion time observed for all other samples. The increase in oxygen is assumed to represent the local area analyzed rather than the entire sample. The EDS elemental analyses therefore suggests that AlAcH treatment with a stronger molar concentration and/or longer immersion time alkali treatment may result in the formation of a more prominent titania layer. The phase of the titania layer cannot be confirmed to be crystalline using EDS however, based on the results presented by Takemoto *et al.* [31] the culminating result of AlAcH treatment is expected to be a crystalline titania layer.

**Table 1:** Chemical composition of the Ti-6Al-4V pre-alloyed powder used in electron beam melting and required by DIN EN ISO 5382-3 standards.

Chemical Element	Al	V	Fe	O	N	H	C	Y	Ti
Used wt.%	6.4	4.1	0.21	0.13	0.01	0.004	0.01	<0.001	Balance
Required wt.%	5.5–6.75	3.5–4.5	<0.3	<0.2	<0.05	<0.015	<0.08	--	Balance

**Table 2:** EDS elemental analysis (wt. %) of AlAcH treated samples for various alkali concentrations and immersion times.

Alkali treatment conditions	O	Al	Ti	V
3M, 6 h	48.89 ± 0.51	2.15 ± 0.14	47.04 ± 0.22	1.91 ± 0.33
5M, 6 h	18.83 ± 1.46	3.88 ± 0.21	74.74 ± 1.72	2.55 ± 0.80
10M, 6 h	23.20 ± 5.35	3.46 ± 0.28	70.88 ± 5.62	2.45 ± 0.15
3M, 24 h	25.66 ± 3.20	4.16 ± 0.17	67.79 ± 3.33	2.38 ± 0.82
5M, 24 h	26.33 ± 2.85	3.30 ± 0.19	68.11 ± 2.90	2.26 ± 0.28
10M, 24 h	28.05 ± 2.48	2.77 ± 0.28	67.58 ± 2.58	1.59 ± 0.04

In conclusion, both SEM and EDS elemental analyses conducted following AlAcH treatment suggest that samples exposed to stronger molar concentration alkali treatment may produce more promising results for apatite forming ability.

### 2.2. Apatite Forming Ability

**Figure 3:**
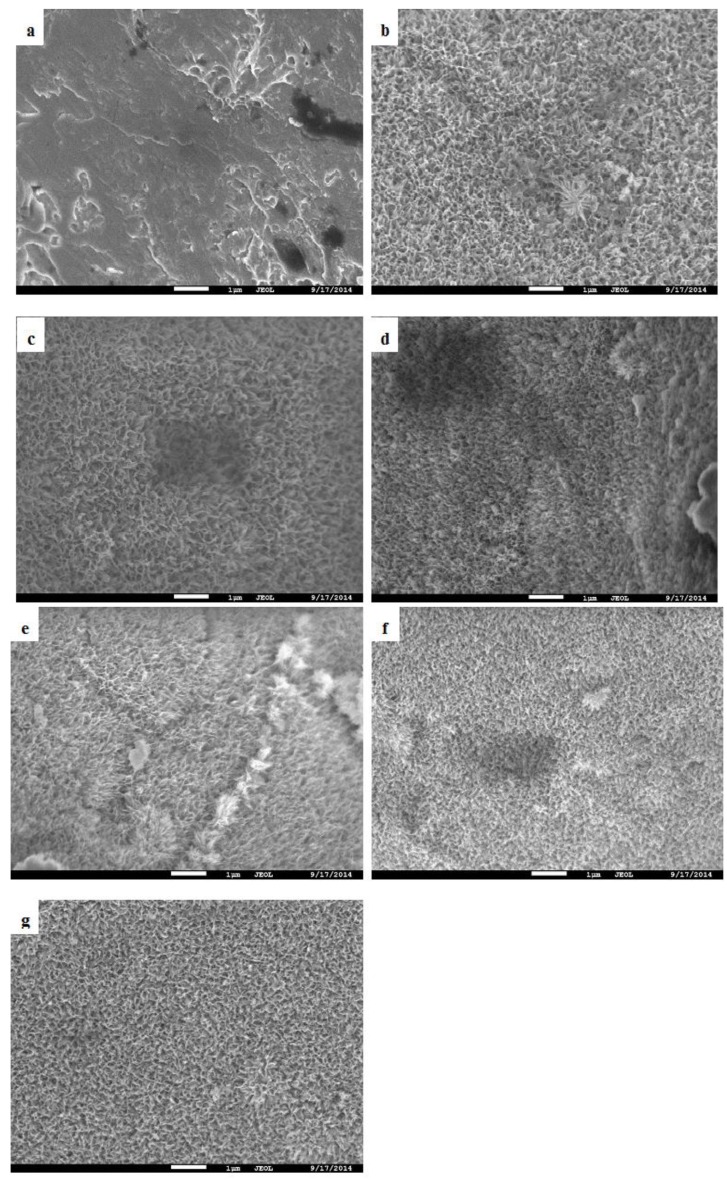
SEM micrographs after 3 week SBF immersion for (**a**) AsM and AlAcH treated samples for alkali treatment (**b**) 3M, 6 h (**c**) 3M, 24 h (**d**) 5M, 6 h (**e**) 5M, 24 h (**f**) 10M, 6 h and (**g**) 10M, 24 h.

**Figure 4:**
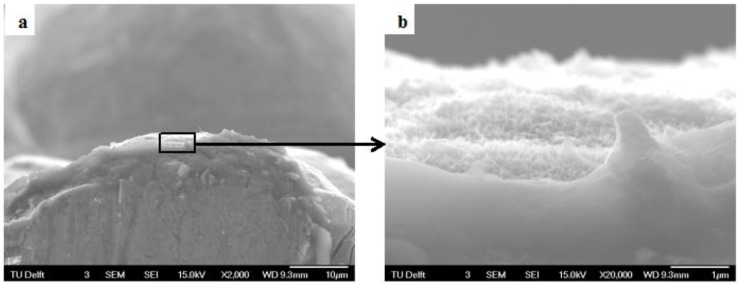
SEM micrograph of cross-sectioned 3M, 6 h AlAcH treated sample at (**a**) 2kX and (**b**) 20kX.

The purpose of chemically treating the porous titanium was to modify its surface topography and chemistry such that its apatite forming ability is improved. The apatite forming ability was tested by immersing AlAcH treated porous titanium samples in SBF for 1 and 3 weeks. After 1 and 3 week immersion in SBF, SEM observations and trends in the EDS results were similar therefore to eliminate redundancy the results following 3 week immersion in SBF are presented and discussed. SEM analysis confirmed that following 3 weeks of SBF immersion all surface topographies were generally unchanged, however, there were a limited number of surface structures that formed on AlAcH treated samples that were distinct to the surrounding topography (Figure 3b,d–f). As previously noted, an increase in nano-topographical feature density was observed with increasing alkali molar concentration and/or immersion time. AsM surfaces remained smooth and featureless after 3 weeks of SBF immersion (Figure 3a). Cross-sections revealed the depth of the nano-features observed on all EBM specimens after surface treatments (Figure 4). In all cases, the depth of the nano-features were in the sub-micron range, measuring a few hundred nanometers in size.

EDS elemental analysis confirmed that the AlAcH treatment was successful in improving Ca and P formation after 3 weeks of immersion in SBF however, no apatite was observed. Although the Ca and P was limited in terms of concentration and in distribution among the surface, both elements were present on all AlAcH treated samples except for those exposed to 3M, 6 h alkali treatment (Table 3). It is assumed that with an alkali treatment of 3M, 6 h the concentration and duration of the alkali treatment were inadequate in forming a substantial sodium titanate layer. Although the samples exposed to 3M, 6 h alkali treatment formed nano-topographical features, the concentration and limited time may have contributed to a lack of a sodium titanate layer and subsequently a lack of Ca formation. Wei *et al.* [39] immersed microarc oxidized TiO2-based films in SBF following 1, 3 and 5M alkali treatment and concluded that apatite formation increased with increasing concentration of NaOH. They reported that the apatite XRD pattern for samples exposed to 3M NaOH for 24 h was very weak while demonstrating strong patterns for 5M alkali treatment. In a similar apatite formation study, Liang *et al.* [40] found that porous titanium fabricated by powder metallurgy exposed to 0.5 or 1M alkali solution had no apatite formation however apatite was observed for those exposed to 5 or 10M alkali solution following SBF testing.

**Table 3:** EDS elemental analysis (wt. %) after 3 week SBF immersion for AsM and AlAcH treated samples with various alkali concentrations and immersion times.

Element	AsM	3M, 6 h	3M, 24 h	5M, 6 h	5M, 24 h	10M, 6 h	10M, 24 h
N	--	9.78 ± 2.91	12.08 ± 7.00	6.15 ± 0.40	0.34 ± 0.59	2.8 ± 2.04	1.20 ± 1.05
O	--	39.80 ± 7.94	31.16 ± 13.84	36.58 ± 9.97	20.07 ± 6.95	21.33 ± 10.92	24.59 ± 8.85
Na	--	--	0.25 ± 0.13	1.11 ± 0.72	3.30 ± 0.62	0.65 ± 0.85	0.12 ± 0.20
Mg	--	--	--	0.01 ± 0.02	0.02 ± 0.03	--	--
Al	4.97 ± 1.83	2.52 ± 0.59	3.08 ± 1.48	2.42 ± 0.07	3.52 ± 0.19	0.73 ± 0.31	0.64 ± 0.11
Si	--	0.03 ± 0.06	0.02 ± 0.03	0.02 ± 0.04	--	--	0.03 ± 0.06
P	--	0.10 ± 0.11	0.20 ± 0.07	0.31 ± 0.01	0.24 ± 0.08	0.29 ± 0.10	0.37 ± 0.23
Cl	--	--	--	0.74 ± 0.13	2.26 ± 0.39	0.11 ± 0.19	--
Ca	--	--	0.13 ± 0.12	0.37 ± 0.01	0.21 ± 0.03	0.15 ± 0.15	0.96 ± 0.13
Ti	90.95 ± 2.22	40.54 ± 10.87	50.97 ± 18.32	50.97 ± 9.63	66.62 ± 7.51	70.28 ± 12.24	69.37 ± 8.39
V	4.08 ± 0.40	1.55 ± 0.56	1.52 ± 0.54	0.69 ± 1.20	2.10 ± 0.07	2.19 ± 0.71	2.58 ± 0.44
Fe	--	5.49 ± 5.76	0.58 ± 0.14	0.61 ± 0.07	1.32 ± 0.23	1.34 ± 0.31	0.16 ± 0.27

In addition, no Ca or P was detected on AsM surfaces following SBF immersion suggesting that the AlAcH treatment does to a degree encourage the formation of both Ca and P as both elements were present on almost all AlAcH treated samples. While there was no distinct pattern in the amount of Ca present based on the molar concentration and/or immersion time of the alkali treatment, wt. % of P was observed to generally increase with increasing molar concentration and immersion time. The greatest amount of Ca and P was observed for samples exposed to 10M, 24 h alkali treatment which coincides with the greatest density of nano-topographical features and presumably the most prominent titania layer. 

The results presented suggest that applying AlAcH treatment does promote Ca and P formation, however, they are preliminary as no apatite formation was visually observed. Nucleation and crystallization of apatite requires the mineral saturation state to be above the equilibrium state, allowing the mineral to precipitate from solution [41,42,43]. Variations in the supersaturated solution caused by circumstances such as an imbalance in SBF reagent concentrations or contamination may hinder nucleation, potentially explaining the lack of apatite formation [44]. Additional parameters of the AlAcH treatment applied should also be adjusted to further optimize the parameters for apatite formation on porous titanium specifically fabricated by EBM. Various other chemical surface treatment studies have shown the apatite forming ability greatly depends on the parameters chosen. For example, while studying alkali-heat surface treatments Uchida *et al.* [45] found that the temperature and immersion time of the water treatment following immersion in NaOH affects the amount of apatite formed. The greatest amount of crystalline titania formed, which they found correlated well with the amount of apatite formation, occurred for the highest temperature and longest immersion time in water (80 °C, 48 h). In a similar study by Kim *et al.* [46], varying the heat treatment temperature following the chemical treatment also affected the apatite forming ability. The heat treatment temperature and duration are significant; it needs to be high and long enough to stabilize the oxide layer but not too high or long as it alters the ratio of anatase/rutile crystalline titania phases which regulate the rate of apatite formation.

Different bioactivity pathways, namely, chemical and biological pathways, should also be considered in analyzing the results [47]. In vitro SBF tests rely on the stimulation of chemical pathways, forming bonds and compounds that stimulate the formation of apatite, and eventually bone regeneration. In vitro cell culture tests also provide chemical pathways as well as bioactivity pathways for bone regeneration through interactions between surface biomolecules and cellular pathways. In some cases the results of SBF and cell culture tests disagree [48], presumably due to differing bioactivity pathways. Amin Yavari *et al.* [28] demonstrated that SLM porous titanium exposed to acid-alkali treatment and porous titanium exposed to an anodizing heat treatment exhibited bioactivity through different pathways. Samples exposed to acid-alkali treatment had the highest apatite forming ability after SBF immersion but performed poorly in cell culture assays with limited cell attachment and proliferation. In contrast, samples exposed to anodizing heat treatment had high levels of cell attachment and proliferation but almost no apatite formation. While their results justify our choice in using SBF as a means of evaluating biofunctionality, the biological bioactivity pathways of differing advanced additive manufacturing techniques, namely SLM and EBM, may differ potentially explaining the lack of apatite formation observed.

The current work does have its limitations. The study is confined in that only the molar concentration and immersion time of the alkali treatment were varied, however, various other parameters such as the temperature, concentration and immersion time of water and acid treatment as well as the temperature and duration of heat treatment should be considered to find ideal parameters for apatite formation. Also, SEM/EDS elemental analysis is limited in characterizing the surface of porous titanium and should only be used for preliminary detection of apatite by visual indication and Ca and P formation; following studies should use X-ray diffraction to detect apatite phase. 

## 3. Experimental Section

### 3.1. Materials and Manufacturing

EBM techniques (implantcast GmbH, Germany) were employed to produce porous titanium samples from Ti6Al4V alloy powder based on DIN EN ISO 5832-3 standards (Table 1) [49]. The samples were produced on a Q10 machine (Arcam AB, Sweden) in a controlled high vacuum chamber kept at 3 × 10E-6 bar using helium gas intake. The porous structures (EPORE^®^) designed and manufactured by implantcast were based on a random structure designed to meet the properties of cancellous bone with strut size of 360 µm, porosity of 60% and specific Young’s Modulus of 3.1 GPa. The samples were discs with an outer diameter of 8 mm, a height of 3 mm and a concentric hole with a diameter of 2 mm.

### 3.2. Surface Treatment 

Prior to surface treatment, all samples were ultrasonically cleaned using ethanol followed by ultrapure water for 10 min each, then were dried in an oven overnight at 40 °C. The samples were then immersed in either 3, 5 or 10M NaOH at 60 °C for either 6 or 24 h as shown in Table 4.

**Table 4:** Alkali treatment parameters tested during AlAcH treatment.

Group name	3M, 6 h	3M, 24 h	5M, 6 h	5M, 24 h	10M, 6 h	10M, 24 h
Molar concentration NaOH (M)	3	3	5	5	10	10
Immersion time (hrs)	6	24	6	24	6	24

After the alkali treatment, the samples were immersed in ultrapure water at 40 °C for 24 h then in 0.5 mM HCl at 40 °C for 24 h. After the acid treatment, the samples were washed with ultrapure water and dried in an oven at 40 °C for 24 h before they were placed in the furnace at 600 °C for 1 h dwelling time. The heating rate of the furnace was 5 °C/min. The samples were allowed to cool down in the furnace. An SEM JEOL (JSM-6500F, Japan) coupled with an EDS was used for observing the surface and conducting elemental analysis. The EDS spectra were taken at an accelerating voltage of 10 kV and a magnification of 12kX.

### 3.3. Apatite Forming Ability

All surface treated samples, along with as manufactured (AsM) samples were immersed in SBF to evaluate their apatite forming ability. The SBF solution was prepared based on NEN-ISO 23317 standards for *in vitro* evaluation for apatite-forming ability of implant materials [50]. 8.035 g NaCl, 0.355 g NaHCO3, 0.225 g KCl, 0.231 g K2HPO4.3H2O, 0.311 g MgCl2.6H2O, 39 mL 1M HCl, 0.292 g CaCl2, 0.072 g Na2SO4 and 6.118 g Tris were added sequentially to 700 mL of deionized water at 37 °C. After dissolving all reagents the pH of the solution was adjusted to 7.4 by incrementally adding 1M HCl. Deionized water was then added to the solution to reach a volume of 1 L.

The samples were individually immersed in 15 mL fresh SBF at 37 °C using 50 mL plastic tubes as described by the standards [46]. The plastic tubes were then placed in a water bath maintained at 37 °C. The samples were removed from the water bath after 1 and 3 weeks, washed with deionized water and dried overnight at 40 °C. An SEM JEOL (JSM-6500F, Japan) coupled with an EDS was used for observing the surface and conducting elemental analysis. The EDS spectra were taken at an accelerating voltage of 10 kV and a magnification of 12kX. To further characterize the surface topography the cross section of samples were also immersed in nitrogen, cross-sectioned and examined by SEM.

## 4. Conclusions

AlAcH treatment successfully modified the topographical and chemical characteristics of EBM porous titanium surface. The chemical and heat treatment created a surface with nano-topographical features ranging in the size of 200–300 nm with a titania layer ideal for apatite formation. After 3 weeks immersion in SBF there was no Ca or P present on the surface of AsM porous titanium, however, both elements were present on all AlAcH treated samples except those exposed to 3M, 6 h alkali treatment. An increase in alkali solution concentration resulted in an increase in the number of nano-topographical features per unit area as well as the amount of titania on the surface. The greatest amount of Ca and P was observed for samples exposed to 10M, 24 h alkali treatment which coincides with the greatest density of nano-topographical features and presumably the most prominent titania layer. Although no apatite was visually observed, the presence of Ca and P does indicate that AlAcH treatment does encourage the formation of both elements as AsM surfaces contained neither. Future studies should focus on optimizing additional parameters in the AlAcH treatment to achieve ideal surface conditions for apatite formation.
